# Anti-Amyloidogenic and Anti-Apoptotic Role of Melatonin in Alzheimer Disease

**DOI:** 10.2174/157015910792246137

**Published:** 2010-09

**Authors:** Hongwen He, Weiguo Dong, Fang Huang

**Affiliations:** Department of Oral Anatomy and Physiology, Guanghua School of Stomatology, Sun Yat-sen University, Guangzhou 510080, P.R. China

**Keywords:** Alzheimer disease, neurodegeneration, neuroprotection, melatonin, amyloid beta-protein, amyloid precursor protein, apoptosis, mitochondria.

## Abstract

Alzheimer disease (AD) is an age-related neurodegenerative disorder characterized by the presence of senile plaques, neurofibrillary tangles and neuronal loss. Amyloid-β protein (Aβ) deposition plays a critical role in the development of AD. It is now generally accepted that massive neuronal death due to apoptosis is a common characteristic in the brains of patients suffering from neurodegenerative diseases, and apoptotic cell death has been found in neurons and glial cells in AD. Melatonin is a secretory product of the pineal gland; melatonin is a potent antioxidant and free radical scavenger and may play an important role in aging and AD. Melatonin decreases during aging and patients with AD have a more profound reduction of this indoleamine. Additionally, the antioxidant properties, the anti-amyloidogenic properties and anti-apoptotic properties of melatonin in AD models have been studied. In this article, we review the anti-amyloidogenic and anti-apoptotic role of melatonin in AD

## 1 INTRODUCTION

	Alzheimer disease (AD), the most common neurodegenerative disease with progressive loss of memory and deterioration of comprehensive cognition, is characterized by senile plaques, neurofibrillary tangles and extensive neuronal loss. These histopathological hallmarks of the disease are observed in the neocortex, hippocampus, and other subcortical regions of AD patient brains; these structures are essential for cognitive function. Amyloid-β protein (Aβ) is the main constituent of senile plaques, which is implicated in the pathogenesis of AD [72]. AD may be further subdivided into early-onset (<65 years old) and late-onset (>65 years old) groups. Notably, patients with either sporadic or familial AD share common clinical and neuropathological markers. Four different genes have been implicated in the etiology of AD: the amyloid precursor protein (APP), apolipoprotein E, and presenilins 1 (PS1) and 2 (PS2) [78, 96].

	Melatonin (N-acetyl-5-methoxytryptamine) is synthesized mainly by the pineal gland during the dark phase of the circadian cycle [83]. Melatonin has a number of physiological functions, including regulating circadian rhythms, clearing free radicals [109, 110], improving immunity, and generally inhibiting the oxidation of biomolecules. Melatonin decreases during the aging process [83, 84] and patients with AD have more profound reductions of this substance [55, 103]. Studies show that melatonin levels are lower in AD patients compared with that in age-matched control subjects [55, 61, 69]. It is generally accepted that a melatonin deficit is closely related to aging and age-related diseases [125]. 

	The great advances have been reported in currently studies of protection against AD by the antioxidant melatonin; this is achieved since melatonin inhibits Aβ-induced toxicity [20, 21, 38, 67, 74, 133] and attenuating tau hyperphosphorylation [14, 45, 54, 56, 118, 119, 121]. Besides the antioxidant properties, the anti-amyloidogenic actions [71, 73] and anti-apoptotic properties [120] of melatonin in AD also have been investigated.

## 2. AMYLOID PRECURSOR PROTEIN, PRESENILINS, AND β-AMYLOID PRODUCTION

Most studies link the pathogenesis of AD with increased production and/or deposition Aβ in the brain. Senile plaques are composed of the Aβ which is a 40-43 amino acid peptide [91, 100, 101]. Aβ is derived from the proteolytic processing of a transmembrane glycoprotein known as β-amyloid precursor protein (APP) [66]. The subsequent cloning of the gene encoding the APP and its localization to chromosome 21 has been achieved [28, 40, 85, 111]. Cleavage of APP at the N-terminus of the Aβ region by β-secretase [114] and at the C-terminus by γ-secretase [88] represents the amyloidogenic pathway for processing of APP to form Aβ. Alternatively, APP can also be processed by α-secretase [113] which cleaves within the Aβ sequence and does not produce Aβ. 

	Mutations in three genes have been linked to early-onset AD. These genes include those encoding for APP, presenilin 1 (PS1) located on chromosome 14 and presenilin 2 (PS2) located on chromosome 1 [52, 95]. More than 100 different mutations in the PS1 gene have been identified [57]. Only a few mutations have been found in the PS2 gene. It seems likely that presenilin proteins either alter the trafficking of APP and its derivatives or they may actually be the γ-secretase responsible for cleavaging of A β from the APP precursor protein [90, 126]. 

	All these mutations lead to an increased production and accumulation of Aβ. On the other hand, the deposition of soluble Aβ produces aggregation of the peptide forming amyloid fibrils which have been reported to be neurotoxic *in vitro* [2, 129] and *in vivo* [35, 102]. These observations led to the amyloid cascade hypothesis which states that excessive production of Aβ is the primary cause of the disease [30]. 

	Amino acid sequencing of the proteins making up cerebral amyloid revealed two common Aβ isoforms. One is termed Aβ40, a 40-amino acid polpeptide, and the other Aβ42, a polypeptide of identical composition but having two additional amino acids at the terminus. Both isoforms of Aβ are hydrophobic and tend to aggregate due to the long stretch of hydrophobic amino acids at the C terminal half of the peptide that forms β-pleated sheet structures characteristic of the Aβ making up the amyloid plaque. In the brains of AD patients, Aβ42 is the predominant species deposited in the brain parenchyma [27]. In contrast, Aβ40 appears to be the predominant species deposited in the cerebral vasculature [37]. Aβ42 is more hydrophobic and aggregates more easily than Aβ40 [44, 123].

## 3. Aβ AND ITS FIBRILLIZATION

Aβ exists in both soluble and fibrillar forms. High levels of fibrillary Aβ are deposited in the AD brain, which is associated with loss of synapses, impairment of neuronal functions and loss of neurons [36, 79, 92, 131]. Formation of Aβ ﬁbrils from soluble Aβ is a multi-step process that is preceded by oligomerization and aggregation of monomeric Aβ, and it involves conformational change of the peptide from α-helical to β-pleated sheet structure [32]. A cascade of metabolic steps begins with the APP protein, its cleavage into Aβ, and the aggregation of Aβ into oligomers, protofibrils, and finally the birefringent amyloid that makes up cerebral plaques [93]. The Aβ oligomeric intermediates (oligomers, protofibrils) and the mature fibrils are all neurotoxic, and it has been demonstrated that the oligomers and protofibrils are actually more neurotoxic than the mature fibrils or amyloid plaques [13]. Extensive evidence shows Aβ ﬁbrils play a causal role in the development of AD-type neuropathology and dementia [24]. Studies with synthetic Aβ have confirmed that Aβ is neurotoxic and that its neurotoxicity is largely dependent on the ability of Aβ to form β-sheet structures or amyloid fibrils [58].

## 4. INHIBITION OF Aβ FIBRIL FORMATION AND Aβ PRODUCTION BY MELATONIN

	The antiamyloidogenic properties of melatonin for AD have been examined [71, 73]. Melatonin pharmacologically reduces normal levels of secretion of soluble APP (sAPP) in different cell lines by interfering with APP full maturation, which would cause a drop in the formation of Aβ itself [46]. Melatonin also affects the mRNA level of APP in a cell type-specific manner. Pretreatment with melatonin resulted in a significant reduction in the APP mRNA level in PC12 cells, but failed to produce this effect in human neuroblastoma cells [99]. In addition, it has been shown that melatonin can interact with Aβ40 and Aβ42 and strongly inhibit the formation of β-sheets and amyloid fibrils *in vitro* [70, 71, 76]. These effects were demonstrated by a number of techniques including circular dichroism, nuclear magnetic resonance spectroscopy and electron microscopy. Skribanek *et al.* [97] also reported that the interaction between Aβ and melatonin was hydrophobic, and took place on the 29-40 residues of the Aβ segment. Pappolla *et al.* [70] further documented a residue-specific interaction between melatonin and any of the three histidine and aspartate residues of Aβ. The imidazole-carboxylate salt bridges formed by the side chains of histidine and aspartate residues play a key role in the formation of the amyloid β-sheet structures [34], and disruption of these salt bridges promotes fibril dissolution [25]. Melatonin may disrupt the imidazole-carboxylate salt bridges and thus prevent Aβ fibrillogenesis and aggregation. This action of melatonin reduces the toxicity of Aβ and also makes it more susceptible to proteolytic degradation. However, melatonin exhibited no significant destabilizing activity toward preformed fAβ1-40 or fAβ1-42 [68]. 

	Wang *et al.* [122] studied the effect of melatonin on Aβ production in wild-type murine neuroblastoma N2a (N2a/wt) and N2a stably transfected with amyloid precursor protein (N2a/APP) cell lines used Sandwich ELISA. The results showed that melatonin suppressed the Aβ level in cell lysates. In addition, melatonin effectively decreased the level of Aβ in N2a/APP [132].

## 5. MELATONIN REDUCES THE AMYLOID BURDEN IN A TRANSGENIC MOUSE MODEL OF AD

A transgenic mouse model for AD mimicking the accumulation of senile plaques, neuronal apoptosis and memory impairment was used in some studies. Moreover, the anti-amyloidogenic role of melatonin was confirmed in a transgenic mouse model of AD. 

Melatonin supplementation in mice led to a significant reduction in levels of toxic cortical Aβ40 and Aβ42 which are involved in amyloid depositions and plaque formation in Alzheimer diseases [47]. Feng *et al.* [19] evaluated the long-term influence of melatonin on neuropathologic changes in APP 695 transgenic mice. Both Congo red staining and Bielschowsky silver impregnation showed that apparent extracellular Aβ deposition in the frontal cortex of APP 695 transgenic mice, but melatonin supplementation inhibited the Aβ deposits.

	Matsubara *et al.* [63] reported that early (starting at 4 months of age) and long-term administration of melatonin partially inhibited the expected time-dependent elevation of Aβ in the treated Tg2576 transgenic mice. Conversly, Qunin *et al.* [81] reported that melatonin failed to modify brain levels of Aβ in Tg2576 transgenic mice that were old enough to have amyloid plaque pathology when treatment was initiated at 14 months of age. Since cortical and hippocampal Aβ continue to accumulate between 14 and 20 months of age, these results indicate that melatonin not only failed to remove existing plaque, but also failed to prevent additional Aβ deposition [80]. The contrary results were because of the age at initiation of treatment. Tg2576 mice in the Matsubara study started melatonin at 4 months of age (prior to the appearance of hippocampal and cortical plaques), compared to 14 months in the Qunin study. Amyloid plaque pathology typically appears in Tg2576 mice at 10-12 months of age [33]. These findings indicate that melatonin has the ability to regulate APP metabolism and prevent Aβ pathology, but fails to exert anti-amyloid or antioxidant effects when initiated after the age of Aβ deposition. In addition, Cheng *et al.* [11] reported that melatonin has differential effects on hippocampal neurodegeneration in different aged SAMP8, the mice initiated treatment from 4-months old exhibited a greater response to melatonin supplementation than 7-months old mice. Melatonin treatment increased hippocampal pyramidal cell number and improved the learning and memory deficits of SAMP8. 

## 6. APOPTOSIS AND AD

Apoptosis is a highly conserved form of cell death that is characterized by chromatin condensation, nuclear fragmentation, cytoplasmic membrane blebbing, and cell shrinkage [41]. Extensive evidence shows that apoptosis is involved in neuronal loss in AD [8, 96, 115]. Postmortem analysis of AD brain shows that there is DNA fragmentation in neurons and glia of hippocampus and cortex as detected by TdT-mediated dUTP nick end labeling (TUNEL) [17, 48, 53, 59, 98, 105, 107]. Increased expression of Bcl-2 family members [16, 26, 43, 60, 65, 106], increased levels of prostate apoptosis response-4 (Par-4) [29], c-Jun protein upregulation [3], increased caspase activities as well as cleavage of caspase substrates have also been detected in AD brain [1, 10, 49, 77, 86, 104, 112, 127]. Moreover, Rohn *et al.* [87] demonstrated the activation of mitochondrial and receptor-mediated apoptotic pathways in AD hippocampal brain sections wherein active caspase-9 was co-localized with active caspase-8. Recently, a marked co-localization of pathological hyperphosphorylated tau, cleaved caspase-3 and caspase-6 have been reported in TUNEL-positive neurons in the brainstem of AD patients [9, 117]. In addition, there is evidence for activation of cell cycle proteins in AD brain [12, 128]. This may be an attempt of the cells to try to survive less than optimal conditions or toxic stimuli [6]. Feng *et al.* [19] reported that cognitive impairment and apoptosis developed in the APP 695 transgenic mice as young as 8 months of age; Apoptosis was most likely contribute to behavioral impairments in the APP 695 transgenic mice. The Aβ can directly induce neuronal apoptosis *in vitro* [31, 75, 89, 124]. Furthermore, *in vitro* studies have shown that Aβ provokes a significant down-regulation of antiapoptotic proteins such as Bcl-2, Bcl-xl and Bcl-w and a significant up-regulation of proapoptotic proteins such as bax [130].

## 7. ANTI-APOPTOTIC ROLE OF MELATONIN IN AD

	*In vitro* experiments showed that Aβ-treated cultures exhibited characteristic features of apoptosis, and melatonin attenuated Aβ-induced apoptosis in a number of cellular models of AD including hippocampal neurons, PC12 cells, mouse microglial BV2 cells and rat astroglioma C6 cells [22, 23, 38, 74, 94].

	Shen *et al.* [94] used Aβ25-35 to induce apoptosis in cultured hippocampal neurons, and monitored the apoptotic activity of the neurons with or without melatonin treatment. The study shows that melatonin at concentrations of 1×10^-6^ and 1×10^-5^ mol/L prevents neuronal morphological changes induced during apoptosis. PC12 cells and rat astroglioma C6 cells treated with either Aβ25-35 or Aβ1-42 underwent apoptosis. Melatonin pretreatment significantly attenuated Aβ25-35 or Aβ1-42-induced apoptosis in PC12 cells and rat astroglioma C6 cells. The anti-apoptotic effects of melatonin were highly reproducible and were corroborated by multiple quantitative methods. In addition, melatonin effectively suppressed Aβ1-42-induced nitric oxide formation, potently prevented Aβ1-40-induced intracellular calcium overload [22, 23]. The experiment in mouse microglial BV2 cells *in vitro* showed that pre-treatment with melatonin in the present study reduced the level of Abeta-induced intracellular ROS (reactive oxygen species) generation, inhibited NF-κB activation, and suppressed the Aβ-induced increase in caspase-3 enzyme activity. In addition, pre-treatment with melatonin inhibits Aβ-induced increase in the levels of Bax mRNA and that it enhances the level of Bcl-2 expression [38]. In addition, melatonin suppresses age-induced apoptosis in cerebellar granule neurons, which may be associated with the activation of Akt, GSK3β and FOXO-1 [108]. *In vivo* experiments suggested that long-term melatonin treatment significantly decreased the TUNEL-positive neurons in APP 695 transgenic mice. 

	There are two major apoptotic signaling pathways in the central nervous system neurodegenerative diseases: extrinsic and intrinsic [120]. The extrinsic apoptotic pathway (death receptor pathway) is initiated by death receptors on the surface of the cells, involving caspase-8/Bid and caspase-10 activation [7, 116, 120]. The intrinsic pathway (the mitochondrial pathway) is involved in the neuroprotection of melatonin [82]. However, there have been no obvious reports of the involvement of extrinsic pathways in the neuroprotection of melatonin. During the progression of neurodegenerative diseases, the survival signaling cascades are activated by neuroprotective agents [64] including the phosphoinositol-3 kinase (PI3K)/Akt pathway [5, 42, 50], the Bcl-2 pathway [82], the NF-κB pathway [18], as well as the MAPK pathway. 

	The highest levels of melatonin are found in the mitochondria [62]. Mitochondria have been identified as a target for melatonin [4, 51]. Melatonin promotes mitochondrial homeostasis. Mitochondria play a critical role in the neuroprotective function of melatonin in AD. Melatonin inhibited the Aβ-induced increase in the levels of mitochondria-related Bax in transgenic AD mice and cultured mouse microglial BV2 cells. Furthermore, *in vivo* observations showed that melatonin-treated animals had diminished expression of NF-κB compared to untreated animals [39]. Furthermore, melatonin prevented upregulated expression of Par-4 and Bax and inhibited Aβ-induced caspase-3 activity [21].

## 8. CONCLUSION

	Melatonin can function as an anti-amyloidogenic and anti-apoptotic indoleamine in addition to having antioxidant properties. Melatonin has been proposed as a treatment for AD. The results from APP transgenic mice have showed that early, long-term melatonin supplementation produces anti-amyloid and antioxidant effects, but no such effect is produced when melatonin treatment is initiated after the age of amyloid formation [47, 63, 80, 132]. The results from SAMP8 have also indicated that differential effects of melatonin on hippocampal neurodegeneration are associated with the age at initiation of treatment [11]. Furthermore, Dong *et al.* [15] reported that melatonin possesses differential effects on Aβ25-35-induced cytotoxicity in hippocampal neurons at different stages of culture, and demonstrated that the differential effects of melatonin were produced through its different actions on mitochondria. These results supported the notion that melatonin or its derived analogs could be explored as a preventive approach in AD, rather than a therapeutic approach. Therefore, extensive clinical trials and studies with transgenic models are necessary to confirm the role of melatonin at the late pathological stage of AD. 
